# Association of lactate-albumin ratio with native liver survival in paediatric acute liver failure: a 10-year retrospective study

**DOI:** 10.1080/07853890.2025.2549135

**Published:** 2025-08-20

**Authors:** Zhen Zhang, Yuna Li, Jingwen Li, Yumei Li

**Affiliations:** Department of PICU, Children’s Medical Center, the First Hospital of Jilin University, Changchun, China

**Keywords:** Lactate–albumin ratio, native liver survival, paediatric acute liver failure

## Abstract

**Background:**

Paediatric acute liver failure (PALF) is a severe life-threatening condition with complex aetiologies and high mortality rates. Prognostic research, particularly in the Chinese paediatric population, is limited. The lactate-albumin ratio (LAR) is a potential prognostic indicator for adverse outcomes in critical illness, and its correlation with survival with the native liver (SNL) in PALF patients requires further study. LAR was calculated as follows: [lactate (mmol/L)/serum albumin (g/L)×100%]. This study utilized early LAR, defined as the initial measurement of lactate and albumin levels conducted within the first 24h following hospital admission, to investigate the correlation between LAR and SNL in PALF.

**Materials and Methods:**

This retrospective cohort study included 77 patients with PALF. The data collected included demographic information, etiologies, complications, laboratory findings, and the Liver Injury Unit score. Logistic regression, smooth curve fitting, generalized additive models, and interaction effects analysis were used for statistical analysis.

**Results:**

Among 77 patients, 46.8% (36 cases) had SNL, with 14.3% (11 cases) requiring transplantation. A significant decrease in SNL was associated with higher LAR (*p* = 0.005), with a 4% decline in SNL for each 1% increase in LAR. When the LAR≥ 10.5%, the odds ratios(ORs) for SNL in Models I, II, and III were 0.259 (95% confidence interval(CI) 0.101-0.668), 0.213 (95% CI 0.076-0.6), and 0.053 (95% CI 0.007-0.378), respectively. Smooth curve fitting confirmed a linear relationship (*p* for nonlinearity = 0.531). The subgroup analysis showed an association between LAR and SNL in non-shocked patients (OR 0.1121, 95% CI 0.0153-0.8206) and significant interactions in patients with higher-grade hepatic encephalopathy (*p* < 0.001). No significant differences in clinical outcomes were observed regardless of etiology.

**Conclusions:**

Our findings suggest a significant correlation between LAR and SNL in patients with PALF from Northeast China. Elevated LAR is predictive of a reduced probability of surviving PALF without the need for liver transplantation, highlighting its potential as a prognostic marker in PALF.

## Introduction

Paediatric acute liver failure (PALF) is a rare and rapidly progressing clinical syndrome [1]. Paediatric Acute Liver Failure (PALF) has varied causes, with acetaminophen overdose common in Western countries and viral infections/metabolic errors more frequent in Asia/Africa. Recent reports also show an increase in unexplained paediatric hepatitis cases [2–5]. PALF outcomes were categorized into three: survival with the native liver (SNL), liver transplantation (LT), and mortality. PALF exhibited a high mortality rate of 40%∼72% in the pre-transplantation era [1,6], which was reduced to 14%∼38% post-transplantation [7]. Accurate prognostic stratification in PALF is crucial for prioritizing emergency LT for patients at a high risk of mortality [8]. Timely prognostic assessment is crucial for enabling prompt clinical decision-making regarding the necessity of emergency LT [9,10]. Despite its recognized importance, precise prognostic evaluation is often challenging in clinical practice [2,11].

Prognostic assessment in PALF is commonly facilitated by prognostic scoring systems and analysis of factors that impact prognosis [12,13]. However, the development and validation of prognostic scores are challenging owing to the rarity of PALF, diversity of aetiologies, and phenotypic heterogeneity of the disease [14–16]. Additionally, research on the correlations among prognostic factors is constrained for the same reasons [1,17,18]. Lactate levels can be elevated due to various reasons, including tissue hypoxia, accelerated glycolysis, and reduced clearance in liver or kidney dysfunction. Elevated serum lactate levels indicate an impaired hepatic clearance capacity, which is commonly observed in PALF [19]. Albumin, a protein synthesized primarily in the liver, is an important marker of hepatic synthetic function. A decrease in serum albumin levels often suggests diminished hepatic synthetic function [20]. The lactate-to-albumin ratio (LAR) combines these two biomarkers, potentially enhancing prognostic accuracy. Previous studies have identified a correlation between hepatic injury and the concentrations of arterial blood lactate and serum albumin [19,21]. Additionally, studies have shown that LAR could serve as a valuable prognostic marker in patients with ACLF (Acute-on-Chronic Liver Failure), with higher LAR values being associated with poorer transplant-free survival and overall survival rates [22]. The concurrent measurement of both lactate and albumin provides a more comprehensive assessment of liver function and the body’s ability to cope with metabolic stress, especially in acute liver failure where both liver synthetic capacity and clearance functions are compromised. However, the assessment of lactate and albumin levels may be constrained by susceptibility to various extraneous factors [23–25]. Studies have shown that the concurrent use of these biomarkers, as represented by the LAR, may enhance predictive accuracy for critical illness over lactate measurement alone [26,27]. To date, comparable studies on PALF are scarce. This study aimed to determine the correlation between early LAR and SNL in PALF to potentially enhance prognostic assessments.

## Materials and methods

### Study population and definition of early LAR

**Figure 1.**
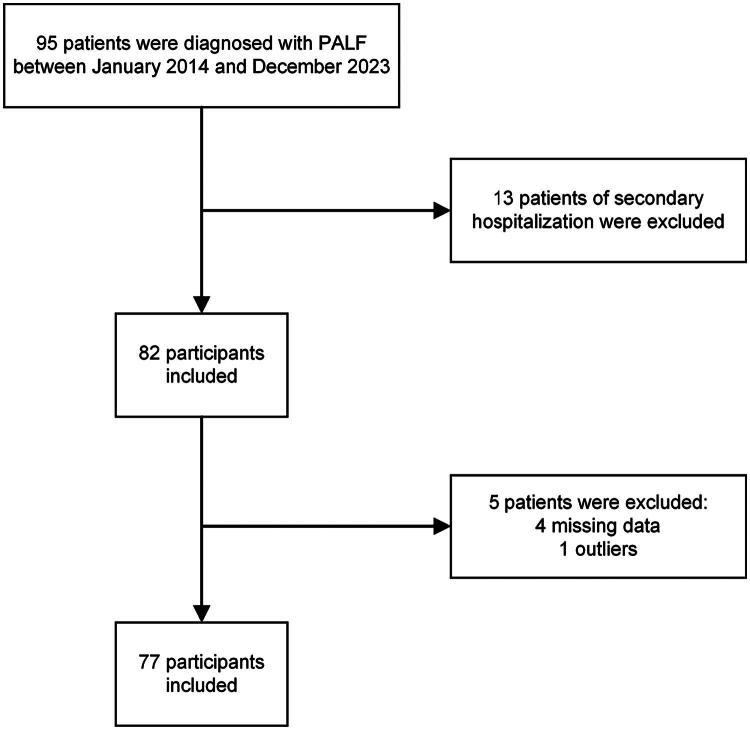
Flowchart.

In this retrospective cohort study, we reviewed the medical records of consecutive patients diagnosed with PALF at the Children’s Medical Center, First Hospital of Jilin University from January 2014 to December 2023. Eligible patients for this study were those ≤18 years of age with a confirmed diagnosis of PALF based on the PALF Study Group (PALFSG) criteria^2^. Patients without lactate or albumin level measurements within the first 24h of hospital admission and/or with ≥10% missing data were excluded from the study. Figure 1 illustrates the exclusion process. Ultimately, 77 patients were included in this study.

LAR was calculated as follows: [lactate (mmol/L)/serum albumin (g/L)×100%]. Early LAR was defined as the initial measurement of lactate and albumin levels conducted within the first 24h following hospital admission. Continuous variables and dichotomous categorizations were employed for data analysis in the context of LAR.

This study was performed in accordance with the Strengthening the Reporting of Observational Studies in Epidemiology guidelines [28] and adhered to the ethical standards of the Helsinki Declaration. The study was approved by the Ethics Committee of the First Hospital of Jilin University. Written/verbal consent was not required because this was a retrospective study and patient data were anonymized prior to analysis.

### Data collection

Patients’ data were obtained from the hospital’s electronic medical records. Demographic, clinical, and laboratory data, as well as information on complications, surgery, and death were extracted. Laboratory data included lactate, albumin, alanine aminotransferase (ALT), aspartate aminotransferase (AST), gamma-glutamyltransferase (GGT), total bilirubin (TBIL), direct bilirubin (DBIL), prothrombin time (PT), international normalized ratio (INR), ammonia (NH_3_), B-type natriuretic peptide (BNP), troponin I (TnI), white blood cell (WBC) count, and platelet count. Liver injury unit(LIU) scores were assessed for each patient. The LIU score was calculated as [3.584 × TBIL(mg/dL)+1.809 × PT(s)+0.307 × NH_3_(μmol/L) [29]. All laboratory data, focused on the initial 24h post-admission, were derived from the first blood sample obtained in the PICU.

### Definitions of early clinical complications

Shock was defined as tissue hypoperfusion accompanied by persistent hypotension (systolic blood pressure below the age-adjusted 5th percentile) or the need for vasoactive support following the administration of ≥20 mL/kg of isotonic fluid resuscitation.

AKI followed the paediatric KDIGO criteria [30].

MODS was diagnosed when dysfunction of ≥ 2 organ systems [31].

HE defined and graded (I-IV) based on the North American Society for Paediatric Gastroenterology, Hepatology, and Nutrition (NASPGHAN) Position Paper for PALF, using criteria involving changes in consciousness, behaviour, and neurologic response, in the absence of other identifiable causes.

### Treatment protocol and liver transplantation

In this study, once PALF was diagnosed, comprehensive medical treatment and artificial liver support therapy were initiated. The artificial liver therapies included continuous renal replacement therapy (CRRT), plasma exchange (PE), and double plasma molecular adsorption system (DPMAS), which were applied alone or in combination based on the patient’s condition. Additionally, the necessity of emergency liver transplantation was assessed according to the LIU score, clinical recommendations, and the overall status of the patient, particularly the severity of hepatic encephalopathy (HE). The selection of the transplantation method was determined based on the willingness of the patient’s relatives to donate a liver and the donor’s medical evaluation, with ethical approval obtained before proceeding with either living donor liver transplantation (LDLT) or donation after circulatory death (DCD) liver transplantation. In this study, all patients underwent artificial liver support therapy, among whom 8 patients received LDLT, and 3 patients underwent DCD liver transplantation.

### Outcomes

The primary endpoint of this study was the outcome at discharge, which included SNL, LT, and mortality. The endpoint for mortality was defined as in-hospital death, excluding cases of death occurring after discharge.

### Statistical analysis

This study aimed to determine the association between LAR and SNL in patients with PALF. Patients were divided into two groups based on their LAR. A descriptive analysis was performed for all patients. Data are expressed as mean ± standard deviation (SD) or median (interquartile range) for continuous variables, and as frequency or percentage for categorical variables. Continuous variables were compared between the groups using Student’s t-test or the Mann–Whitney U-test, depending on the normality of the distribution. Fisher’s exact test was used to compare categorical variables between groups. Multivariable logistic regression models were built to adjust for potential confounders in the association between LAR and SNL, which were shown as odds ratios (ORs) with 95% confidence intervals (CIs). Both the non-adjusted and multivariate models were used. Multivariate models included factors of clinical interest and significant covariates in the univariate analysis. Other potential confounders were chosen based on previous scientific data or a change > 10% in the effect estimates. LAR was utilized as both a continuous and categorical variable in the logistic regression models, with a trend test conducted. We created three models: Model I was not adjusted for confounders, Model II was adjusted for age and sex, and Model III was additionally adjusted for reasons, acute kidney injury (AKI), HE, ALT, PT, NH_3_, TnI, Platelet, and LIU score. Smooth curve fitting and a generalized additive model(GAM) were used to examine the associations between LAR and SNL. The level of missing data for all variables in the regression models was 5%, and imputation methods were employed when the proportion exceeded this threshold.

In addition, a sensitivity analysis was performed to improve the robustness of the results. Based on the above results, patients with lower LAR levels were selected as the reference group. Analyses were stratified according to age, sex, complications and LIU score. Interactions across subgroups were tested using a likelihood ratio test.

All statistical analyses were performed using R Statistical Software (Version 4.2.2, http://www.R-project.org, The R Foundation) and the Free Statistics analysis platform (Version 1.9, Beijing, China, http://www.clinicalscientists.cn/freestatistics). Statistical significance was defined as a two-sided *p* value < 0.05.

## Results

### Baseline characteristics of patients by LAR

A total of 77 patients admitted to the PICU due to PALF were included in our study. The descriptive characteristics of the eligible study population are presented in Table 1. The median age was 72 months (range, 2–168 months), and 39 patients (50.6%) were male. Among all patients, 36 (46.8%) had SNL, 11 (14.3%) underwent LT, and 30 (39%) had fatal outcomes. All the LT patients were discharged. The median LIU score was 111.56 (range, 45.054–454.198). The median LAR was 10.5% (range, 2.2%–101%). For analysis, LAR was categorized using the median value of 10.5% as the cutoff, based on the distribution within our study cohort. This threshold was data-driven and not derived from predefined clinical criteria. 39 (50.6%) patients presented with LAR ≥10.5%. In patients with LAR ≥10.5%, the prevalence of SNL was 30.8%, which was significantly lower than the 63.2% in those with LAR <10.5% (*p* < 0.001). Additionally, higher LAR was significantly associated with adverse outcomes (*p* < 0.001), including LT or mortality, complications such as shock (*p* = 0.044) multiple organ dysfunction syndrome (MODS) (*p* = 0.029), and elevated BNP levels (*p* = 0.004). However, no statistically significant differences were observed with respect to age, sex or etiological classification. A detailed comparison of baseline clinical and laboratory variables between the SNL(+) and SNL(–) groups is presented in Supplementary Table 1. Patients in the SNL(–) group were more likely to experience severe HE (≥grade 3), shock, and MODS. In the SNL(–) group, LAR values were significantly higher than in the SNL(+) group (median 15.7% vs. 9.2%, *p* = 0.019).

**Table 1.** Baseline epidemiological and clinical characteristics.

Variables	Total (*n* = 77)	Binary variable		*p*_value
		LAR < 10.5 (*n* = 38)	LAR ≥ 10.5 (*n* = 39)	
Sex, n (%)				0.087
male	39 (50.6)	23 (60.5)	16 (41)	
female	38 (49.4)	15 (39.5)	23 (59)	
Age(month)	72.0 (12.0, 120.0)	60.0 (12.0, 105.0)	84.0 (18.5, 120.0)	0.438
Aetiology, n (%)				0.728
infectious	22 (28.6)	10 (26.3)	12 (30.8)	
toxic	10 (13.0)	7 (18.4)	3 (7.7)	
idiopathic	24 (31.2)	11 (28.9)	13 (33.3)	
metabolic	14 (18.2)	7 (18.4)	7 (17.9)	
tumours and others	7 (9.1)	3 (7.9)	4 (10.3)	
AKI, n (%)				0.817
No	62 (80.5)	31 (81.6)	31 (79.5)	
Yes	15 (19.5)	7 (18.4)	8 (20.5)	
HE, n (%)				0.162
<grade 3	53 (68.8)	29 (76.3)	24 (61.5)	
≥grade 3	24 (31.2)	9 (23.7)	15 (38.5)	
Shock, n (%)				0.044
No	57 (74.0)	32 (84.2)	25 (64.1)	
Yes	20 (26.0)	6 (15.8)	14 (35.9)	
MODS, n (%)				0.029
No	31 (40.3)	20 (52.6)	11 (28.2)	
Yes	46 (59.7)	18 (47.4)	28 (71.8)	
Outcome, n (%)				0.001
LT	11 (14.3)	7 (18.4)	4 (10.3)	
death	30 (39.0)	7 (18.4)	23 (59)	
SNL	36 (46.8)	24 (63.2)	12 (30.8)	
LIU score	111.6 (91.2, 158.7)	110.3 (93.4, 132.4)	112.5 (91.1, 179.3)	0.473
ALT (U/L)	515.1 (99.6, 1820.6)	542.2 (131.0, 1410.0)	515.1 (77.5, 1833.3)	0.879
AST (U/L)	434.0 (166.7, 1435.8)	319.4 (109.3, 1242.5)	473.0 (190.6, 1610.4)	0.343
GGT (U/L)	53.5 (29.6, 91.4)	65.5 (36.8, 88.7)	44.7 (25.3, 95.6)	0.485
TBIL (μmol/L)	129.0 (36.5, 248.5)	130.9 (47.0, 241.9)	105.0 (26.0, 233.2)	0.992
DBIL (μmol/L)	54.1 (18.1, 130.2)	79.4 (20.8, 131.7)	50.7 (17.3, 125.5)	0.764
Albumin (g/L)	31.5 (25.0, 36.0)	34.6 (30.3, 36.8)	28.8 (21.9, 33.0)	0.001
PT (s)	26.8 (22.6, 37.8)	25.8 (20.5, 33.2)	29.2 (22.9, 42.3)	0.142
INR	2.4 (1.9, 3.3)	2.2 (1.8, 2.9)	2.6 (1.9, 3.6)	0.16
NH_3_ (μmol/L)	78.0 (53.0, 164.0)	70.5 (50.5, 124.8)	87.0 (59.0, 166.5)	0.436
BNP (ng/mL)	647.0 (150.0, 3310.0)	262.0 (129.0, 1947.5)	1520.0 (375.5, 5360.0)	0.004
TnI (ng/mL)	0.02 (0.01, 0.31)	0.01 (0.01, 0.29)	0.02 (0.01, 0.33)	0.166
Lactate (mmol/L)	3.2 (2.2, 5.2)	2.2 (1.7, 3.1)	5.2 (4.1, 8.9)	< 0.001
WBC (×10^9^/L)	11.8 (7.1, 17.6)	9.9 (6.8, 14.8)	12.2 (8.1, 22.2)	0.119
Platelet (×10^9^/L)	192.0 (88.0, 307.0)	200.0 (80.8, 329.2)	182.0 (105.0, 245.5)	0.839
LAR (%)	10.5 (7.3, 19.6)	7.2 (5.6, 8.9)	19.6 (16.0, 33.9)	< 0.001

Note: Data are presented as count (percentage) or median (IQR).

Abbreviations: AKI, acute kidney injury; ALT, alanine aminotransferase; AST, aspartate aminotransferase; BNP, B-type natriuretic peptide; DBIL, direct bilirubin; GGT, gamma-glutamyltransferase; HE, hepatic encephalopathy; INR, international normalized ratio; IQR, interquartile range; LAR, lactate-albumin ratio; LIU, liver injury unit; LT, liver transplantation; MODS, multiple organ dysfunction syndrome; NH_3_, ammonia; PT, prothrombin time; SNL, survival with the native liver; TBIL, total bilirubin; TnI, troponin I; WBC, white blood cell.

### Association between LAR and SNL

Univariate and multivariate logistic regression analyses demonstrated a significant association between LAR and SNL in PALF patients. In univariate analysis (Table 2), LAR was associated with SNL (OR 0.9606, 95%CI 0.9237–0.999, *p* = 0.04465) when analyzed as a continuous variable, and this association was strengthened when LAR was dichotomized into a binary variable (OR 0.2593, 95%CI 0.1006-0.6684, *p* = 0.00521).

**Table 2.** Association of covariates and survival with the native liver.

Variable	OR_95CI	*p*_value
Sex: female	1.5972 (0.6484 ∼ 3.9343)	0.30862
Age	0.9943 (0.9858 ∼ 1.0029)	0.19006
Aetiology: toxic	0.4286 (0.0874 ∼ 2.1014)	0.29624
Aetiology: idiopathic	1.4 (0.4367 ∼ 4.4879)	0.57131
Aetiology: metabolic	0.75 (0.1947 ∼ 2.8893)	0.67589
Aetiology: tumours and others	0.4 (0.0635 ∼ 2.52)	0.32918
AKI	1.3878 (0.4478 ∼ 4.3004)	0.57012
HE ≥ grade 3	0.1868 (0.0605 ∼ 0.5761)	0.00351
Shock	0.2796 (0.0895 ∼ 0.8728)	0.02822
MODS	0.2304 (0.0871 ∼ 0.6098)	0.00311
LIU score	0.9958 (0.9893 ∼ 1.0024)	0.21071
ALT	1.0001 (0.9999 ∼ 1.0002)	0.42279
AST	1 (0.9999 ∼ 1.0001)	0.73263
GGT	1.0024 (0.9961 ∼ 1.0087)	0.44936
TBIL	0.9998 (0.9974 ∼ 1.0022)	0.84952
DBIL	0.9998 (0.9958 ∼ 1.0038)	0.91396
PT	0.9922 (0.9746 ∼ 1.0101)	0.39257
INR	0.891 (0.7249 ∼ 1.0953)	0.27334
NH_3_	0.9935 (0.9868 ∼ 1.0003)	0.0602
BNP	1 (1 ∼ 1)	0.37608
TnI	0.4775 (0.2014 ∼ 1.1322)	0.09334
WBC	0.9743 (0.932 ∼ 1.0185)	0.24968
Platelet	1.0028 (0.9996 ∼ 1.0059)	0.08168
Lactate	0.8842 (0.7687 ∼ 1.0171)	0.08487
Albumin	1.0637 (0.9935 ∼ 1.1389)	0.07637
LAR(%)	0.9606 (0.9237 ∼ 0.999)	0.04465
LAR ≥ 10.5%	0.2593 (0.1006 ∼ 0.6684)	0.00521

Abbreviations: AKI, acute kidney injury; ALT, alanine aminotransferase; AST, aspartate aminotransferase; BNP, B-type natriuretic peptide; DBIL, direct bilirubin; GGT, gamma-glutamyltransferase; HE, hepatic encephalopathy; INR, international normalized ratio; LAR, lactate-albumin ratio; LIU, liver injury unit; MODS, multiple organ dysfunction syndrome; NH_3_, ammonia; PT, prothrombin time; SNL, survival of the native liver; TBIL, total bilirubin; TnI, troponin I; WBC, white blood cell.

Additionally, HE grade 3 or higher (OR 0.1868, 95% CI 0.0605-0.5761, *p* = 0.00351), shock (OR 0.2796, 95% CI 0.0895–0.8728, *p* = 0.02822) and MODS (OR 0.2304, 95% CI 0.0871–0.6098, *p* = 0.00311) were significantly associated with SNL (Table 2). The results of the multivariate regression analysis are shown in Table 3. An association was revealed in all three models between LAR and SNL (ORs range 0.932-0.961). This association was further strengthened when LAR was dichotomized into a binary variable; patients with LAR ≥10.5% had a 74.1% reduced rate of SNL compared with those with LAR <10.5% (OR = 0.259, 95%CI 0.101-0.668). After adjusting for all covariates, the results showed a 94.7% reduced rate of SNL in patients with an LAR ≥ 10.5% (OR = 0.053, 95% CI 0.007-0.378) than in those with LAR < 10.5%.

**Table 3.** Multivariate logistic regression for lactate-albumin ratio on survival with the native liver.

Variable	LAR(%)		Binary variable		
			LAR < 10.5%	LAR ≥ 10.5%	
	OR (95%CI)	*p*_value	OR(95%CI)	OR(95%CI)	*p*_value
Model I	0.961 (0.924 ∼ 0.999)	0.0446	1(Ref)	0.259 (0.101 ∼ 0.668)	0.0052
Model II	0.96 (0.923 ∼ 0.998)	0.0409	1(Ref)	0.213 (0.076 ∼ 0.6)	0.0034
Model III	0.932 (0.877 ∼ 0.99)	0.0219	1(Ref)	0.053 (0.007 ∼ 0.378)	0.0034

**Abbreviations:** AKI, acute kidney injury; ALT, alanine aminotransferase; HE: hepatic encephalopathy; LAR, lactate-albumin ratio; LIU, liver injury unit; NH_3_, ammonia; PT, prothrombin time; SNL, survival of the native liver; TnI, troponin I.

Model I: No adjusted.

Model II: Adjusted for sex + age.

Model III: adjusted for Model II + reasons + AKI + HE + ALT + PT + NH_3_ + TnI + Platelet + LIU score.

**Figure 2.**
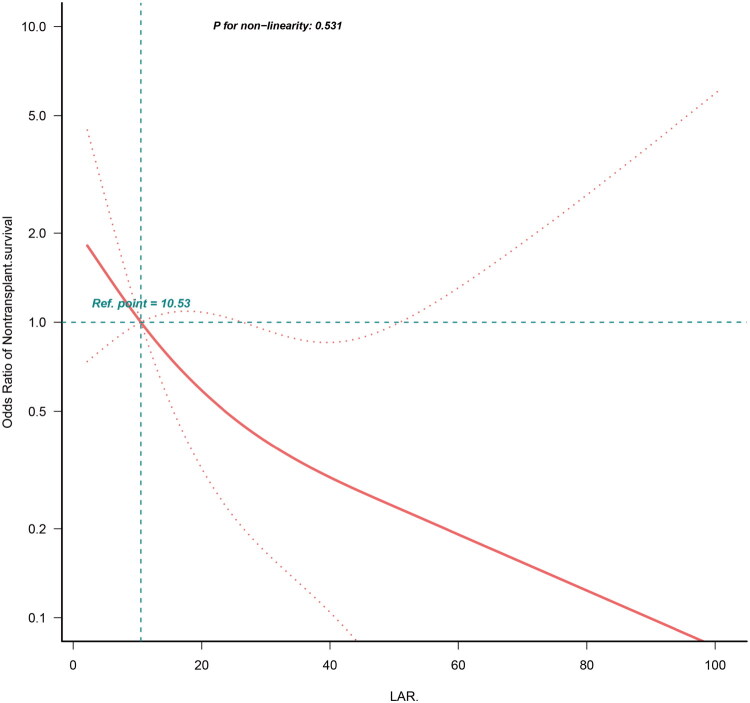
Restricted cubic splines model reveals a negative linear relationship between lactate-albumin ratio and survival with the native liver. **Abbreviations:** LAR, lactate-albumin ratio; SNL, survival with the native liver

**Figure 3.**
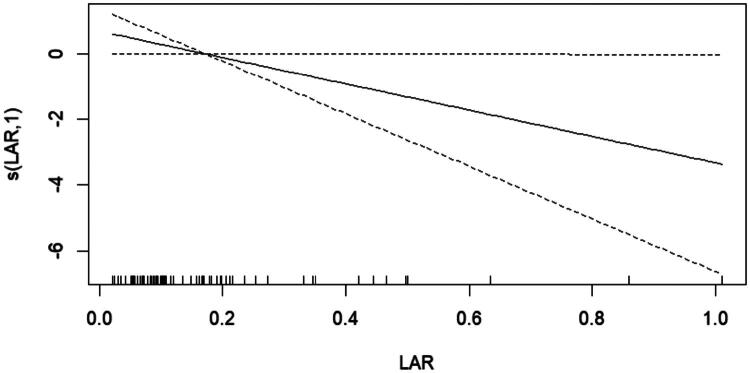
Generalized additive model reveals a negative linear relationship between lactate-albumin ratio and survival with the native liver. **Abbreviations:** LAR, lactate-albumin ratio; s(LAR,1): survival with the native liver

Using the GAM, we found that the relationship between LAR and SNL was linear (Figure 2). Similar results were observed in the restricted cubic spline (RCS) model (*p* for non-linearity = 0.531) (Figure 3).

### Subgroup analyses

Subgroup analyses were conducted to evaluate the impact of LAR on SNL, stratified by age, sex, AKI, HE, shock, and LIU score, using multivariate logistic regression models. The results of subgroup analyses are shown in Table 4. LAR was associated with SNL in patients without shock (OR 0.1121; 95% CI 0.0153–0.8206). No such association was observed in other subgroups. Significant interactions were identified within the subgroup of patients with HE of grade ≥3 (*p* < 0.001), with no such interactions noted in other subgroups.

**Table 4.** Stratified multivariable analysis of the association between lactate-albumin ratio and survival with the native liver.

Subgroup	Variable	n.total	n.event_%	OR_95CI	*p*_value	*p*.for.interaction
**Sex**						0.791
male						
	LAR < 10.5%	23	12 (52.2)	1(Ref)		
	LAR ≥ 10.5%	16	4 (25)	0 (0∼Inf)	0.99983	
female						
	LAR < 10.5%	15	12 (80)	1(Ref)		
	LAR ≥ 10.5%	23	8 (34.8)	0 (0∼Inf)	0.99895	
**Age**						0.096
<6 y						
	LAR < 10.5%	19	15 (78.9)	1(Ref)		
	LAR ≥ 10.5%	19	4 (21.1)	0 (0∼Inf)	0.99979	
≥6y						
	LAR < 10.5%	19	9 (47.4)	1(Ref)		
	LAR ≥ 10.5%	20	8 (40)	3.942 × 10^48^ (0∼Inf)	0.9988	
**AKI**						0.388
No						
	LAR < 10.5%	31	19 (61.3)	1(Ref)		
	LAR ≥ 10.5%	31	9 (29)	0.3778 (0.0371 ∼ 3.8518)	0.41129	
Yes						
	LAR < 10.5%	7	5 (71.4)	1(Ref)		
	LAR ≥ 10.5%	8	3 (37.5)	0 (0∼Inf)	0.9999	
**HE**						<0.001
<grade 3						
	LAR < 10.5%	29	19 (65.5)	1(Ref)		
	LAR ≥ 10.5%	24	12 (50)	12.9501 (0.1239 ∼ 1353.4427)	0.28028	
≥grade 3						
	LAR < 10.5%	9	5 (55.6)	1(Ref)		
	LAR ≥ 10.5%	15	0 (0)	0 (0∼Inf)	0.9999	
**Shock**						0.362
No						
	LAR < 10.5%	32	22 (68.8)	1(Ref)		
	LAR ≥ 10.5%	25	9 (36)	0.1121 (0.0153 ∼ 0.8206)	0.03119	
Yes						
	LAR < 10.5%	6	2 (33.3)	1(Ref)		
	LAR ≥ 10.5%	14	3 (21.4)	0 (0∼Inf)	0.99995	
**LIU score**						0.181
≤110.87						
	LAR < 10.5%	20	14 (70)	1(Ref)		
	LAR ≥ 10.5%	18	5 (27.8)	0 (0∼Inf)	0.99934	
≥111.56						
	LAR < 10.5%	18	10 (55.6)	1(Ref)		
	LAR ≥ 10.5%	21	7 (33.3)	0 (0∼Inf)	0.99895	

**Abbreviations:** AKI, acute kidney injury; ALT, alanine aminotransferase; HE, hepatic encephalopathy; LAR, lactate-albumin ratio; LIU, liver injury unit; MODS, multiple organ dysfunction syndrome; NH_3_, ammonia; SNL, survival of the native liver; TnI, troponin I.

Adjusted for sex + reasons + AKI + HE + shock + MODS + ALT + PT+NH_3_+TnI + Platelet + LIU score.

### Aetiology and outcome

The aetiologies of PALF were categorized into five classes: infection (28.6%), intoxication (13%), metabolic disorders (18.2%), unknown causes (31.2%), and neoplasms along with other causes (9.1%). The outcomes of SNL, liver transplantation, and mortality across different aetiologies are shown in Table 5.

**Table 5.** Aetiology and outcome.

Variables	Total (*n* = 77)	SNL (*n* = 36)	LT (*n* = 11)	death (*n* = 30)	*p*
Aetiology, n (%)					0.172
infectious	22 (28.6)	11 (30.6)	1 (9.1)	10 (33.3)	
toxic	10 (13.0)	3 (8.3)	3 (27.3)	4 (13.3)	
idiopathic	24 (31.2)	14 (38.9)	1 (9.1)	9 (30)	
metabolic	14 (18.2)	6 (16.7)	3 (27.3)	5 (16.7)	
tumours and others	7 (9.1)	2 (5.6)	3 (27.3)	2 (6.7)	

## Discussion

PALF is an acute and rapidly progressing clinical condition with poor prognosis that requires time-sensitive decision-making. Despite acknowledging the importance of risk stratification in PALF, satisfactory tools for this purpose are lacking. Thus, research into the risk factors associated with the prognosis of PALF would significantly contribute to decision-making. Our study is the first to assess the efficacy of early LAR in patients with PALF, demonstrating a significant association between early LAR and SNL in PALF. After adjusting for potential confounders, this association remained significant. When early LAR was treated as a categorical variable, a more significant difference was observed compared with the continuous variable. In the subgroup analysis, a stronger correlation between LAR and SNL was evident among non-shock patients. No significant variations in clinical outcomes were observed across the diverse aetiologies of PALF.

In our univariate regression analysis, neither lactate nor albumin showed significant associations with SNL when evaluated independently. However, LAR, computed from the combination of these variables, exhibited a statistically significant correlation with SNL (Table 2). This suggests that LAR may serve as a more sensitive prognostic indicator of SNL than either lactate or albumin alone. Several studies have indicated that lactate levels may serve as a potential independent risk factor for the severity and adverse prognosis of liver disease. Bhakta et al. demonstrated that the inclusion of lactate in the model for end-stage liver disease (MELD) score enhanced the predictive accuracy for the prognosis of cirrhosis [32]. In our study, lactate levels were substantially elevated above the normal threshold (2 mmol/L) in all patients, with a median value of 3.2 mmol/L (range, 0.5–21.8 mmol/L). These levels exhibited a negative correlation with SNL, although this correlation was not statistically significant. These observations suggest that elevated lactate levels may serve as a marker of PALF. However, the prognostic value of lactate as a sole biomarker appears to be constrained. The liver is the primary site for albumin synthesis, making it a true marker of hepatic synthetic function. Some studies have suggested a role for albumin in chronic liver disease. However, owing to its long half-life, serum albumin levels may not reflect acute changes in acute liver disease compared with chronic liver disease. In our study, univariate analysis confirmed the proposed association, revealing a positive correlation between serum albumin level and SNL. However, this correlation was not statistically significant (OR 1.0637, 95% CI 0.9935–1.1389). Consequently, the prognostic value of albumin was enhanced when it was combined with lactate levels. This prognostic value was particularly evident when albumin and lactate levels were analysed together as dichotomous variables, revealing a statistically significant difference. In our multivariate regression analysis, the modelling outcomes revealed a negative correlation between LAR and SNL. Following the adjustment for potential confounders, this relationship remained stable. In a stratified analysis, when LAR ≥ 10.5%, the trend corresponded to that observed with the continuous variable, with highly pronounced statistical significance. These results imply that LAR may act as an independent prognostic factor for adverse outcomes in PALF patients.

LAR, initially described by Wang et al. in 2015, has been suggested as a prognostic indicator of mortality and onset of organ dysfunction [33]. Subsequent research has shown a correlation between increased LAR and poor outcomes in various diseases including sepsis, AKI, and pancreatitis [34–36]. A study utilizing the eICU database revealed that LAR was significantly associated with both hospital and ICU mortality (adjusted hazard ratio [HR] 1.22, 95% CI 1.18-1.26, *p* < 0.0001 for both). These findings indicate that elevated LAR is an independent risk factor for in-hospital and intensive care unit (ICU) mortality among critically ill patients with AKI [36].

An additional study that enrolled 648 critically ill children in paediatric intensive care units (PICUs) throughout the United States demonstrated that early implementation of LAR was significantly correlated with mortality and the onset of MODS, compared to the individual initial measurements of lactate or albumin alone [26]. However, studies investigating the relationship between LAR and liver disease are more common in adults, while they are scarce in children. Several studies have shown that LAR is an effective prognostic marker in cirrhotic and ACLF patients. Elevated LAR is significantly associated with in-hospital mortality and liver-related adverse outcomes, with similar prognostic value to traditional scoring systems such as APACHE II and SOFA scores [37–39].

Although most prior studies on LAR have focused on chronic liver disease or critical illness, its use in PALF is pathophysiologically justified. In PALF, LAR likely reflects both acute hepatic dysfunction and systemic stress: elevated lactate suggests impaired clearance and mitochondrial failure, while low albumin indicates reduced synthesis, inflammation, and may also reflect capillary leakage. Unlike in sepsis or ACLF, where LAR reflects circulatory failure, in PALF it may represent overall metabolic crisis and liver reserve. Although a high LAR was inversely associated with SNL, we interpret it broadly as a marker of poor prognosis, rather than distinguishing between death and transplant—consistent with our study’s focus on native liver survival.

We also acknowledge that treatment-related factors prior to or during early PICU admission may influence initial LAR values. For instance, the use of vasoactive agents such as epinephrine can increase lactate levels independently of tissue hypoxia, thereby potentially confounding the association between LAR and clinical outcomes. Although detailed data on drug types and dosages were unavailable, we performed a stratified analysis and found that the association between high LAR and reduced SNL was significant only in the non-shock subgroup. This suggests that LAR is more robust as a prognostic marker when not influenced by shock-related hyperlactatemia.

Furthermore, in the present study, both the GAM and RCS models revealed a linear correlation between LAR and SNL. This implies that in clinical settings, an elevated LAR is associated with a greater likelihood of adverse outcomes, such as heightened mortality or the need for liver transplantation, corroborating the findings of prior studies [35,36].

In the subgroup analysis, LAR was found to be correlated with SNL in patients who did not experience shock. As is well known, the pathophysiology of shock is characterized by inadequate tissue perfusion, which is a primary contributor to the excessive production of lactate. A significant correlation between the LAR and SNL was evident when the confounding effects of tissue hypoperfusion on lactate levels were excluded. Consequently, shock, with its signature tissue hypoperfusion, has emerged as a significant confounder in the relationship between LAR and SNL. Although we recognize that the constrained sample size within the subgroup analysis may impact the robustness of the findings, these results are congruent with the established pathophysiological mechanisms of the disease.

This study offers robust evidence supporting the relationship between LAR and SNL in patients with PALF, meticulously accounting for potential confounders and bias. However, this study had several limitations. First, this was a single-center retrospective analysis. Some cases were excluded due to missing early albumin data, which may introduce selection bias. To improve data completeness and study robustness, we are actively planning to integrate institutional databases and conduct prospective cohort studies. Second, the study’s limited sample size restricted its ability to conduct a comprehensive evaluation of statistical power and investigate interactions among various factors. Given the rarity of PALF, the small population size represents a significant limitation not only for the current study but also for similar research. Nonetheless, our study successfully enrolled a larger cohort of participants from a single institution than previous studies, and it provides detailed insights into the clinical characteristics of PALF in the Northeast region of China, which have not been previously described. Finally, in order to minimize the influence of confounding variables, we chose to use the initial measurements within the first 24 h of hospital admission, thereby avoiding the potential impact of subsequent therapeutic interventions on the study outcomes. However, this approach also precludes the assessment of the dynamic effects of LAR changes on clinical prognosis. In future studies, we plan to consider performing multiple lactate measurements at different time points (such as at admission, at 6 h, peak lactate, and lactate clearance) and use these dynamic changes to calculate the lactate-albumin ratio. This will help provide a more comprehensive evaluation of the clinical significance of the lactate-albumin ratio as a prognostic marker.

## Conclusion

In summary, this study revealed a significant negative correlation between early LAR and SNL in patients with PALF, indicating that early LAR may be a more reliable indicator of SNL than either initial lactate or initial albumin considered in isolation. LAR demonstrated a markedly stronger association with SNL than when initial lactate or albumin levels were evaluated alone. The results of this study have implications for clinical decision-making and the prognostic evaluation of PALF. Acknowledging the potential for confounding factors, additional research is warranted to confirm these findings and deepen our understanding of this relationship.

## Acknowledgements

We extend our heartfelt gratitude to all the participants for their invaluable contributions to this study. Special thanks are due to Dr. Jie Liu from the Department of Vascular and Endovascular Surgery at the Chinese PLA General Hospital for his invaluable assistance in the study design, language editing, proofreading, statistical support, and insightful comments on the manuscript.

## Supplementary Material

Supplementary Table 1.docx

Figure_legends.docx

STROBE Checklist.docx

Manuscript document.docx

## Data Availability

The raw data necessary to replicate the findings of this study cannot be shared at this time as they are part of an ongoing study. Upon request, some or all data generated or utilized during the study can be obtained from the corresponding author.
