# Supplementary material for: Association of lactate-albumin ratio with native liver survival in paediatric acute liver failure: a 10-year retrospective study
Source: Ann Med. 2025 Aug 20;57(1):2549135. doi: 10.1080/07853890.2025.2549135 (PMC12369520; doi:10.1080/07853890.2025.2549135)
Supplement: Supplementary Table 1.docx [file IANN_A_2549135_SM1584.docx]

Supplementary Table 1 Comparison between SNL(+) and SNL(–) groups

| Variables | Total (n = 77) | SNL(–) (n = 41) | SNL(+) (n = 36) | p |
| --- | --- | --- | --- | --- |
| Sex, n (%) |  |  |  | 0.307 |
| male | 39 (50.6) | 23 (56.1) | 16 (44.4) |  |
| female | 38 (49.4) | 18 (43.9) | 20 (55.6) |  |
| Age(month) | 72.0 (12.0, 120.0) | 84.0 (24.0, 120.0) | 48.0 (12.0, 87.0) | 0.167 |
| Etiology, n (%) |  |  |  | 0.487 |
| infectious | 22 (28.6) | 11 (26.8) | 11 (30.6) |  |
| toxic | 10 (13.0) | 7 (17.1) | 3 (8.3) |  |
| idiopathic | 24 (31.2) | 10 (24.4) | 14 (38.9) |  |
| metabolic | 14 (18.2) | 8 (19.5) | 6 (16.7) |  |
| tumors and others | 7 ( 9.1) | 5 (12.2) | 2 (5.6) |  |
| AKI, n (%) |  |  |  | 0.569 |
| No | 62 (80.5) | 34 (82.9) | 28 (77.8) |  |
| Yes | 15 (19.5) | 7 (17.1) | 8 (22.2) |  |
| HE, n (%) |  |  |  | 0.002 |
| <grade 3 | 53 (68.8) | 22 (53.7) | 31 (86.1) |  |
| ≥grade 3 | 24 (31.2) | 19 (46.3) | 5 (13.9) |  |
| shock, n (%) |  |  |  | 0.023 |
| No | 57 (74.0) | 26 (63.4) | 31 (86.1) |  |
| Yes | 20 (26.0) | 15 (36.6) | 5 (13.9) |  |
| MODS, n (%) |  |  |  | 0.002 |
| No | 31 (40.3) | 10 (24.4) | 21 (58.3) |  |
| Yes | 46 (59.7) | 31 (75.6) | 15 (41.7) |  |
| LIU score | 111.6 (91.2, 158.7) | 118.8 (92.1, 183.9) | 109.4 (90.3, 127.8) | 0.195 |
| ALT (U/L) | 515.1 (99.6, 1820.6) | 390.0 (66.5, 1189.3) | 937.9 (210.0, 3358.6) | 0.112 |
| AST (U/L) | 434.0 (166.7, 1435.8) | 431.0 (166.7, 1435.8) | 611.4 (158.2, 1405.0) | 0.61 |
| GGT (U/L) | 53.5 (29.6, 91.4) | 44.7 (26.6, 85.9) | 73.8 (35.4, 95.9) | 0.097 |
| TBIL (μmol/L) | 129.0 (36.5, 248.5) | 129.0 (25.6, 215.3) | 116.6 (47.3, 249.1) | 0.87 |
| DBIL (μmol/L) | 54.1 (18.1, 130.2) | 57.4 (13.9, 130.2) | 53.4 (22.9, 135.2) | 0.706 |
| Albumin (g/L) | 31.5 (25.0, 36.0) | 30.5 (22.3, 34.4) | 32.2 (28.7, 36.5) | 0.086 |
| PT (s) | 26.8 (22.6, 37.8) | 29.2 (22.6, 41.1) | 25.8 (22.6, 33.9) | 0.481 |
| INR | 2.4 (1.9, 3.3) | 2.6 (1.9, 3.5) | 2.2 (1.9, 2.9) | 0.366 |
| NH_3_ (μmol/L) | 78.0 (53.0, 164.0) | 114.0 (59.0, 169.0) | 66.0 (50.2, 115.0) | 0.09 |
| BNP (ng/mL) | 647.0 (150.0, 3310.0) | 1280.0 (323.0, 3840.0) | 262.0 (138.2, 1900.0) | 0.045 |
| TnI (ng/mL) | 0.0 (0.0, 0.3) | 0.0 (0.0, 0.6) | 0.0 (0.0, 0.1) | 0.022 |
| Lactate (mmol/L) | 3.2 (2.2, 5.2) | 4.3 (2.5, 7.2) | 3.0 (2.2, 3.5) | 0.04 |
| WBC (×10^9^/L) | 11.8 (7.1, 17.6) | 11.4 (7.0, 21.9) | 11.9 (7.3, 14.8) | 0.603 |
| Platelet (×10^9^/L) | 192.0 (88.0, 307.0) | 162.0 (83.0, 238.0) | 213.0 (98.5, 374.0) | 0.068 |
| LAR (%) | 10.5 (7.3, 19.6) | 15.7 (8.9, 21.5) | 9.2 (6.9, 12.1) | 0.019 |

Note: Data are presented as count (percentage) or median (IQR).

Abbreviations: SNL, survival with the native liver; AKI, acute kidney injury; ALT, alanine aminotransferase; AST, aspartate aminotransferase; BNP, B-type natriuretic peptide; DBIL, direct bilirubin; GGT, gamma-glutamyltransferase; HE, hepatic encephalopathy; INR, international normalized ratio; IQR, interquartile range; LAR, lactate-albumin ratio; LIU, liver injury unit; LT, liver transplantation; MODS, multiple organ dysfunction syndrome; NH_3_, ammonia; PT, prothrombin time; SNL, survival with the native liver; TBIL, total bilirubin; TnI, troponin I; WBC, white blood cell.
