# Supplementary material for: Association of lactate-albumin ratio with native liver survival in paediatric acute liver failure: a 10-year retrospective study
Source: Ann Med. 2025 Aug 20;57(1):2549135. doi: 10.1080/07853890.2025.2549135 (PMC12369520; doi:10.1080/07853890.2025.2549135)
Supplement: Figure_legends.docx [file IANN_A_2549135_SM1583.docx]

Figure 1 Flowchart

Figure 2 Restricted cubic splines model reveals a negative linear relationship between lactate-albumin ratio and survival with the native liver

**Abbreviations:** LAR, lactate-albumin ratio; SNL, survival with the native liver

Figure 3 Generalized additive model reveals a negative linear relationship between lactate-albumin ratio and survival with the native liver

**Abbreviations:** LAR, lactate-albumin ratio; s(LAR,1): survival with the native liver
