# Supplementary material for: Association of lactate-albumin ratio with native liver survival in paediatric acute liver failure: a 10-year retrospective study
Source: Ann Med. 2025 Aug 20;57(1):2549135. doi: 10.1080/07853890.2025.2549135 (PMC12369520; doi:10.1080/07853890.2025.2549135)
Supplement: STROBE Checklist.docx [file IANN_A_2549135_SM1582.docx]

**STROBE Checklist for Cohort Studies**

| Item | Recommendation | Location in Manuscript / Notes |
| --- | --- | --- |
| Title and Abstract |  |  |
| 1a | Indicate the study’s design with a commonly used term in the title or the abstract | Title includes “A Ten‑Year Retrospective Study”, clearly specifying a retrospective cohort design (Title Page). |
| 1b | Provide in the abstract an informative and balanced summary of what was done and what was found | Structured abstract summarises study setting, cohort size (n = 77), exposure (early LAR), outcome (SNL/LT/death), statistical methods, and key findings (page 1–2). |
| Introduction |  |  |
| 2 | Explain the scientific background and rationale for the investigation being reported | Introduction paragraphs 1‑3 outline limited prognostic tools in PALF and biological rationale for using LAR (pages 3–5). |
| 3 | State specific objectives, including any prespecified hypotheses | Last paragraph of Introduction states objective: to assess association between early LAR and SNL; hypothesis that higher LAR predicts reduced SNL (page 5). |
| Methods |  |  |
| 4 | Present key elements of study design early in the paper | Methods > Study Population: retrospective cohort of PALF admissions 2014‑2023 (page 5). |
| 5 | Describe the setting, locations, and relevant dates, including periods of recruitment, exposure, follow-up, and data collection | Department of PICU, Children’s Medical Center, the First Hospital of Jilin University, Changchun, China; Jan 2014‑Dec 2023 (page 5). |
| 6a | Give the eligibility criteria, and the sources and methods of selection of participants. Describe methods of follow-up | Eligible patients for this study were those ≤18 years of age with a confirmed diagnosis of PALF based on the PALF Study Group (PALFSG) criteria2. Patients without lactate or albumin level measurements within the first 24h of hospital admission and/or with ≥10% missing data were excluded from the study. |
| 6b | For matched studies, give matching criteria and number of exposed and unexposed | Not applicable – no matching. |
| 7 | Clearly define all outcomes, exposures, predictors, potential confounders, and effect modifiers. Give diagnostic criteria, if applicable | Exposure: early LAR (continuous & dichotomised). Outcome: SNL at discharge. Covariates: age, sex, reasons, AKI, HE, ALT, PT, NH3, TnI, Platelet, LIU score (page 6‑8). |
| 8 | For each variable of interest, give sources of data and details of methods of assessment (measurement). Describe comparability of assessment methods if there is more than one group | Laboratory data from first blood sample within 24 h; clinical data from electronic records; definitions provided under Data Collection (page 6‑7). |
| 9 | Describe any efforts to address potential sources of bias | Consecutive inclusion to reduce selection bias; multivariable adjustment & subgroup analyses to control confounding (page 8-9). |
| 10 | Explain how the study size was arrived at | All eligible PALF patients over 10‑year period were included (n = 77); no formal sample‑size calculation (page 5). |
| 11 | Explain how quantitative variables were handled in the analyses. If applicable, describe which groupings were chosen and why | LAR analysed as categorical (≥ median 10.5 % vs < 10.5 %); rationale given (page 10). |
| 12a | Describe all statistical methods, including those used to control for confounding | Univariate & multivariable logistic regression, GAM, restricted cubic splines; confounders selected a priori and by >10 % change‑in‑estimate rule (page 8‑9). |
| 12b | Describe any methods used to examine subgroups and interactions | Subgroup analyses were performed using multivariable logistic regression models stratified by age, sex, AKI, HE, shock, and LIU score. Interaction effects were tested using likelihood ratio tests comparing models with and without the interaction terms (page 19-21). |
| 12c | Explain how missing data were addressed | Variables with >5 % missing were imputed (details page 9). |
| 12d | If applicable, explain how loss to follow‑up was addressed | Not applicable – outcomes assessed at discharge; no loss to follow‑up. |
| 12e | Describe any sensitivity analyses | Sensitivity analysis using LAR<10.5 % as reference across subgroups (page 10). |
| Results |  |  |
| 13a | Report numbers of individuals at each stage of the study | 77 eligible patients included; flowchart illustrates screening, inclusion/exclusion (Figure 1). |
| 13b | Give reasons for non‑participation at each stage | Excluded: absent early lactate/albumin or extreme outlier data (Figure 1). |
| 13c | Consider use of a flow diagram | Flow diagram provided (Figure 1). |
| 14a | Give characteristics of study participants (eg demographic, clinical, social) and information on exposures and potential confounders | Baseline demographics, clinical variables in Table 1; supplementary Table 1 for SNL groups. |
| 14b | Indicate number of participants with missing data for each variable of interest | 4 patients lacked albumin measurements within the first 24 hours of PICU admission. |
| 14c | Summarise follow‑up time (e.g., average and total amount) | Not applicable – cross‑section up to discharge. |
| 15 | Report numbers of outcome events or summary measures over time | Outcome counts: SNL = 36, LT = 11, death = 30 (Results section, Table 1). |
| 16a | Give unadjusted estimates and, if applicable, confounder‑adjusted estimates and their precision (eg, 95% confidence interval). Make clear which confounders were adjusted for and why they were included | Tables 2‑3 present unadjusted and adjusted ORs with 95 %CIs for LAR on SNL. |
| 16b | Report category boundaries when continuous variables were categorised | Median LAR 10.5 % used as cut‑point (Results, page 10). |
| 16c | If relevant, consider translating estimates of relative risk into absolute risk for a meaningful time period | Not performed; outcomes already reported as absolute numbers. |
| 17 | Report other analyses done – e.g., analyses of subgroups and interactions, and sensitivity analyses | Subgroup & interaction analyses in Table 4; GAM and RCS plots in Figures 2‑3. |
| Discussion |  |  |
| 18 | Summarise key results with reference to study objectives | First paragraph of Discussion restates objective and key finding that higher early LAR predicts lower SNL (page 22). |
| 19 | Discuss limitations of the study, taking into account sources of potential bias or imprecision. Discuss both direction and magnitude of any potential bias | Limitations paragraph addresses retrospective design, single‑centre setting, missing data, small sample (page 27). |
| 20 | Give a cautious overall interpretation of results considering objectives, limitations, multiplicity of analyses, results from similar studies, and other relevant evidence | Interpretation paragraphs compare with adult data on LAR, discuss biological plausibility and implications (pages 22‑27). |
| 21 | Discuss the generalisability (external validity) of the study results | This study was a single-center retrospective study, and its findings require further validation through large-scale, multicenter, prospective studies to ensure their reliability and generalizability. |
| Other Information |  |  |
| 22 | Give the source of funding and the role of the funders for the present study and, if applicable, for the original study on which the present article is based | Funding statement on Funding Details section: Jilin Province grants; funders had no role. |

Prepared for submission with the manuscript “Association of Lactate-Albumin Ratio with Native Liver Survival in Pediatric Acute Liver Failure: A Ten-Year Retrospective Study”.
