# Supplementary material for: Association of lactate-albumin ratio with native liver survival in paediatric acute liver failure: a 10-year retrospective study
Source: Ann Med. 2025 Aug 20;57(1):2549135. doi: 10.1080/07853890.2025.2549135 (PMC12369520; doi:10.1080/07853890.2025.2549135)
Supplement: Manuscript document.docx [file IANN_A_2549135_SM1581.docx]

**TITLE PAGE**

**Title:** Association of Lactate-Albumin Ratio with Native Liver Survival in Pediatric Acute Liver Failure: A Ten-Year Retrospective Study

Zhen Zhang^1^, M.D., Yuna Li ^2^, M.M., Jingwen Li^3^, M.M., Yumei Li ^4*^, M.D.

^1234^Department of PICU, Children's Medical Center, the First Hospital of Jilin University, Changchun, China

^*^ Corresponding author: Yumei Li, M.D. E-mail address: [ym_li@jlu.edu.cn](mailto:ym_li@jlu.edu.cn)

Postal address: 1 Xinmin Street, Changchun, Jilin Province, China

Phone: 86-15804302907

Zhen Zhang ([zhangzhen@jlu.edu.cn](mailto:ym_li@jlu.edu.cn)),

Yuna Li (yuna23@jlu.edu.cn)

Jingwen Li (957425476@qq.com)

Yumei Li ([ym_li@jlu.edu.cn](mailto:ym_li@jlu.edu.cn))

## Abstract

**Background:** Pediatric acute liver failure (PALF) is a severe life-threatening condition with complex etiologies and high mortality rates. Prognostic research, particularly in the Chinese pediatric population, is limited. The lactate-albumin ratio (LAR) is a potential prognostic indicator for adverse outcomes in critical illness, and its correlation with survival with the native liver (SNL) in PALF patients requires further study. LAR was calculated as follows: [lactate (mmol/L)/serum albumin (g/L)×100%]. This study utilized early LAR, defined as the initial measurement of lactate and albumin levels conducted within the first 24h following hospital admission, to investigate the correlation between LAR and SNL in PALF. **Materials and Methods:** This retrospective cohort study included 77 patients with PALF. The data collected included demographic information, etiologies, complications, laboratory findings, and the Liver Injury Unit score. Logistic regression, smooth curve fitting, generalized additive models, and interaction effects analysis were used for statistical analysis. **Results:** Among 77 patients, 46.8% (36 cases) had SNL, with 14.3% (11 cases) requiring transplantation. A significant decrease in SNL was associated with higher LAR (*p* = 0.005), with a 4% decline in SNL for each 1% increase in LAR. When the LAR≥ 10.5%, the odds ratios(ORs) for SNL in Models I, II, and III were 0.259 (95% confidence interval(CI) 0.101-0.668), 0.213 (95% CI 0.076-0.6), and 0.053 (95% CI 0.007-0.378), respectively. Smooth curve fitting confirmed a linear relationship (*p* for nonlinearity=0.531). The subgroup analysis showed an association between LAR and SNL in non-shocked patients (OR 0.1121, 95% CI 0.0153-0.8206) and significant interactions in patients with higher-grade hepatic encephalopathy (*p*<0.001). No significant differences in clinical outcomes were observed regardless of etiology. **Conclusions:** Our findings suggest a significant correlation between LAR and SNL in patients with PALF from Northeast China. Elevated LAR is predictive of a reduced probability of surviving PALF without the need for liver transplantation, highlighting its potential as a prognostic marker in PALF.

## Keywords

lactate-albumin ratio, native liver survival, pediatric acute liver failure

## Introduction

Pediatric acute liver failure (PALF) is a rare and rapidly progressing clinical syndrome [1]. Pediatric Acute Liver Failure (PALF) has varied causes, with acetaminophen overdose common in Western countries and viral infections/metabolic errors more frequent in Asia/Africa. Recent reports also show an increase in unexplained pediatric hepatitis cases [2-5]. PALF outcomes were categorized into three: survival with the native liver (SNL), liver transplantation (LT), and mortality. PALF exhibited a high mortality rate of 40%~72% in the pretransplantation era [1, 6], which was reduced to 14%~38% posttransplantation [7]. Accurate prognostic stratification in PALF is crucial for prioritizing emergency LT for patients at a high risk of mortality [8]. Timely prognostic assessment is crucial for enabling prompt clinical decision making regarding the necessity of emergency LT [9, 10]. Despite its recognized importance, precise prognostic evaluation is often challenging in clinical practice [2, 11].

Prognostic assessment in PALF is commonly facilitated by prognostic scoring systems and analysis of factors that impact prognosis [12, 13]. However, the development and validation of prognostic scores are challenging owing to the rarity of PALF, diversity of etiologies, and phenotypic heterogeneity of the disease [14-16]. Additionally, research on the correlations among prognostic factors is constrained for the same reasons [1, 17, 18]. Lactate levels can be elevated due to various reasons, including tissue hypoxia, accelerated glycolysis, and reduced clearance in liver or kidney dysfunction. Elevated serum lactate levels indicate an impaired hepatic clearance capacity, which is commonly observed in PALF [19]. Albumin, a protein synthesized primarily in the liver, is an important marker of hepatic synthetic function. A decrease in serum albumin levels often suggests diminished hepatic synthetic function [20]. The lactate-to-albumin ratio (LAR) combines these two biomarkers, potentially enhancing prognostic accuracy. Previous studies have identified a correlation between hepatic injury and the concentrations of arterial blood lactate and serum albumin [19, 21]. Additionally, studies have shown that LAR could serve as a valuable prognostic marker in patients with ACLF (Acute-on-Chronic Liver Failure), with higher LAR values being associated with poorer transplant-free survival and overall survival rates [22]. The concurrent measurement of both lactate and albumin provides a more comprehensive assessment of liver function and the body's ability to cope with metabolic stress, especially in acute liver failure where both liver synthetic capacity and clearance functions are compromised. However, the assessment of lactate and albumin levels may be constrained by susceptibility to various extraneous factors [23-25]. Studies have shown that the concurrent use of these biomarkers, as represented by the LAR, may enhance predictive accuracy for critical illness over lactate measurement alone [26, 27]. To date, comparable studies on PALF are scarce. This study aimed to determine the correlation between early LAR and SNL in PALF to potentially enhance prognostic assessments.

## Materials and Methods

### Study population and definition of early LAR

In this retrospective cohort study, we reviewed the medical records of consecutive patients diagnosed with PALF at the Children's Medical Center, First Hospital of Jilin University from January 2014 to December 2023. Eligible patients for this study were those ≤18 years of age with a confirmed diagnosis of PALF based on the PALF Study Group (PALFSG) criteria^2^. Patients without lactate or albumin level measurements within the first 24h of hospital admission and/or with ≥10% missing data were excluded from the study. **Figure 1** illustrates the exclusion process. Ultimately, 77 patients were included in this study.

LAR was calculated as follows: [lactate (mmol/L)/serum albumin (g/L)×100%]. Early LAR was defined as the initial measurement of lactate and albumin levels conducted within the first 24h following hospital admission. Continuous variables and dichotomous categorizations were employed for data analysis in the context of LAR.

This study was performed in accordance with the Strengthening the Reporting of Observational Studies in Epidemiology guidelines [28] and adhered to the ethical standards of the Helsinki Declaration. The study was approved by the Ethics Committee of the First Hospital of Jilin University. Written/verbal consent was not required because this was a retrospective study and patient data were anonymized prior to analysis.

### Data collection

Patients’ data were obtained from the hospital’s electronic medical records. Demographic, clinical, and laboratory data, as well as information on complications, surgery and death were extracted. Laboratory data included lactate, albumin, alanine aminotransferase (ALT), aspartate aminotransferase (AST), gamma-glutamyltransferase (GGT), total bilirubin (TBIL), direct bilirubin (DBIL), prothrombin time (PT), international normalized ratio (INR), ammonia (NH_3_), B-type natriuretic peptide (BNP), troponin I (TnI), white blood cell (WBC) count, and platelet count. Liver injury unit(LIU) scores were assessed for each patient. The LIU score was calculated as [3.584×TBIL(mg/dL)+1.809×PT(s)+0.307×NH_3_(μmol/L) [29]. All laboratory data, focused on the initial 24h post-admission, were derived from the first blood sample obtained in the PICU.

### Definitions of Early Clinical Complications

Shock was defined as tissue hypoperfusion accompanied by persistent hypotension (systolic blood pressure below the age-adjusted 5th percentile) or the need for vasoactive support following the administration of ≥20 mL/kg of isotonic fluid resuscitation.

AKI followed the pediatric KDIGO criteria [30].

MODS was diagnosed when dysfunction of≥2 organ systems [31].

HE defined and graded (I-IV) based on the North American Society for Pediatric Gastroenterology, Hepatology, and Nutrition (NASPGHAN) Position Paper for PALF, using criteria involving changes in consciousness, behavior, and neurologic response, in the absence of other identifiable causes.

### Treatment Protocol and Liver Transplantation

In this study, once PALF was diagnosed, comprehensive medical treatment and artificial liver support therapy were initiated. The artificial liver therapies included continuous renal replacement therapy (CRRT), plasma exchange (PE), and double plasma molecular adsorption system (DPMAS), which were applied alone or in combination based on the patient’s condition. Additionally, the necessity of emergency liver transplantation was assessed according to the LIU score, clinical recommendations, and the overall status of the patient, particularly the severity of hepatic encephalopathy (HE). The selection of the transplantation method was determined based on the willingness of the patient's relatives to donate a liver and the donor's medical evaluation, with ethical approval obtained before proceeding with either living donor liver transplantation (LDLT) or donation after circulatory death (DCD) liver transplantation. In this study, all patients underwent artificial liver support therapy, among whom 8 patients received LDLT, and 3 patients underwent DCD liver transplantation.

### Outcomes

The primary endpoint of this study was the outcome at discharge, which included SNL, LT, and mortality. The endpoint for mortality was defined as in-hospital death, excluding cases of death occurring after discharge.

### Statistical analysis

This study aimed to determine the association between LAR and SNL in patients with PALF. Patients were divided into two groups based on their LAR. A descriptive analysis was performed for all patients. Data are expressed as mean±standard deviation (SD) or median (interquartile range) for continuous variables, and as frequency or percentage for categorical variables. Continuous variables were compared between the groups using Student’s t-test or the Mann–Whitney U test, depending on the normality of the distribution. Fisher’s exact test was used to compare categorical variables between groups. Multivariable logistic regression models were built to adjust for potential confounders in the association between LAR and SNL, which were shown as odds ratios (ORs) with 95% confidence intervals (CIs). Both the non-adjusted and multivariate models were used. Multivariate models included factors of clinical interest and significant covariates in the univariate analysis. Other potential confounders were chosen based on previous scientific data or a change > 10% in the effect estimates. LAR was utilized as both a continuous and categorical variable in the logistic regression models, with a trend test conducted. We created three models: Model I was not adjusted for confounders, Model II was adjusted for age and sex, and Model III was additionally adjusted for reasons, acute kidney injury (AKI), HE, ALT, PT, NH_3_, TnI, Platelet and LIU score. Smooth curve fitting and a generalized additive model(GAM) were used to examine the associations between LAR and SNL. The level of missing data for all variables in the regression models was 5%, and imputation methods were employed when the proportion exceeded this threshold.

In addition, a sensitivity analysis was performed to improve the robustness of the results. Based on the above results, patients with lower LAR levels were selected as the reference group. Analyses were stratified according to age, sex, complications and LIU score. Interactions across subgroups were tested using a likelihood ratio test.

All statistical analyses were performed using R Statistical Software (Version 4.2.2, http://www.R-project.org, The R Foundation) and the Free Statistics analysis platform (Version 1.9, Beijing, China, http://www.clinicalscientists.cn/freestatistics). Statistical significance was defined as a two-sided *p* value<0.05.

## Results

### Baseline characteristics of patients by LAR

A total of 77 patients admitted to the PICU due to PALF were included in our study. The descriptive characteristics of the eligible study population are presented in **Table 1**. The median age was 72 months (range, 2-168 months), and 39 patients (50.6%) were male. Among all patients, 36 (46.8%) had SNL, 11 (14.3%) underwent LT, and 30 (39%) had fatal outcomes. All the LT patients were discharged. The median LIU score was 111.56 (range, 45.054-454.198). The median LAR was 10.5% (range, 2.2%-101%). For analysis, LAR was categorized using the median value of 10.5% as the cutoff, based on the distribution within our study cohort. This threshold was data-driven and not derived from predefined clinical criteria. 39 (50.6%) patients presented with LAR ≥10.5%. In patients with LAR ≥10.5%, the prevalence of SNL was 30.8%, which was significantly lower than the 63.2% in those with LAR <10.5% (*p*<0.001). Additionally, higher LAR was significantly associated with adverse outcomes (*p*<0.001), including LT or mortality, complications such as shock (*p*=0.044) multiple organ dysfunction syndrome (MODS) (*p*=0.029), and elevated BNP levels (*p*=0.004). However, no statistically significant differences were observed with respect to age, sex or etiological classification. A detailed comparison of baseline clinical and laboratory variables between the SNL(+) and SNL(–) groups is presented in Supplementary Table 1. Patients in the SNL(–) group were more likely to experience severe HE (≥grade 3), shock, and MODS. In the SNL(–) group, LAR values were significantly higher than in the SNL(+) group (median 15.7% vs. 9.2%, p = 0.019).

### Association between LAR and SNL

Univariate and multivariate logistic regression analyses demonstrated a significant association between LAR and SNL in PALF patients. In univariate analysis (**Table 2**), LAR was associated with SNL (OR 0.9606, 95%CI 0.9237-0.999, *p*=0.04465) when analyzed as a continuous variable, and this association was strengthened when LAR was dichotomized into a binary variable (OR 0.2593, 95%CI 0.1006-0.6684, *p*=0.00521).

Additionally, HE grade 3 or higher (OR 0.1868, 95% CI 0.0605-0.5761, *p*=0.00351), shock (OR 0.2796, 95% CI 0.0895-0.8728, *p*=0.02822) and MODS (OR 0.2304, 95% CI 0.0871-0.6098, *p*=0.00311) were significantly associated with SNL (**Table 2**). The results of the multivariate regression analysis are shown in **Table 3**. An association was revealed in all three models between LAR and SNL (ORs range 0.932-0.961). This association was further strengthened when LAR was dichotomized into a binary variable; patients with LAR ≥10.5% had a 74.1% reduced rate of SNL compared with those with LAR <10.5% (OR=0.259, 95%CI 0.101-0.668). After adjusting for all covariates, the results showed a 94.7% reduced rate of SNL in patients with an LAR≥10.5% (OR=0.053, 95% CI 0.007-0.378) than in those with LAR<10.5%.

Using the GAM, we found that the relationship between LAR and SNL was linear (**Figure 2**). Similar results were observed in the restricted cubic spline (RCS) model (*p* for non-linearity = 0.531) (**Figure 3**).

### Subgroup analyses

Subgroup analyses were conducted to evaluate the impact of LAR on SNL, stratified by age, sex, AKI, HE, shock, and LIU score, using multivariate logistic regression models. The results of subgroup analyses are shown in **Table 4**. LAR was associated with SNL in patients without shock(OR 0.1121；95% CI 0.0153-0.8206). No such association was observed in other subgroups. Significant interactions were identified within the subgroup of patients with HE of grade ≥3 (*p* < 0.001), with no such interactions noted in other subgroups.

### Etiology and outcome

The etiologies of PALF were categorized into five classes: infection (28.6%), intoxication (13%), metabolic disorders (18.2%), unknown causes (31.2%), and neoplasms along with other causes (9.1%). The outcomes of SNL, liver transplantation, and mortality across different etiologies are shown in **Table 5**.

## Discussion

PALF is an acute and rapidly progressing clinical condition with poor prognosis that requires time-sensitive decision-making. Despite acknowledging the importance of risk stratification in PALF, satisfactory tools for this purpose are lacking. Thus, research into the risk factors associated with the prognosis of PALF would significantly contribute to decision-making. Our study is the first to assess the efficacy of early LAR in patients with PALF, demonstrating a significant association between early LAR and SNL in PALF. After adjusting for potential confounders, this association remained significant. When early LAR was treated as a categorical variable, a more significant difference was observed compared with the continuous variable. In the subgroup analysis, a stronger correlation between LAR and SNL was evident among non-shock patients. No significant variations in clinical outcomes were observed across the diverse etiologies of PALF.

In our univariate regression analysis, neither lactate nor albumin showed significant associations with SNL when evaluated independently. However, LAR, computed from the combination of these variables, exhibited a statistically significant correlation with SNL (Table 2). This suggests that LAR may serve as a more sensitive prognostic indicator of SNL than either lactate or albumin alone. Several studies have indicated that lactate levels may serve as a potential independent risk factor for the severity and adverse prognosis of liver disease. Bhakta et al. demonstrated that the inclusion of lactate in the model for end-stage liver disease (MELD) score enhanced the predictive accuracy for the prognosis of cirrhosis [32]. In our study, lactate levels were substantially elevated above the normal threshold (2 mmol/L) in all patients, with a median value of 3.2 mmol/L (range, 0.5-21.8 mmol/L). These levels exhibited a negative correlation with SNL, although this correlation was not statistically significant. These observations suggest that elevated lactate levels may serve as a marker of PALF. However, the prognostic value of lactate as a sole biomarker appears to be constrained. The liver is the primary site for albumin synthesis, making it a true marker of hepatic synthetic function. Some studies have suggested a role for albumin in chronic liver disease. However, owing to its long half-life, serum albumin levels may not reflect acute changes in acute liver disease compared with chronic liver disease. In our study, univariate analysis confirmed the proposed association, revealing a positive correlation between serum albumin level and SNL. However, this correlation was not statistically significant (OR 1.0637, 95% CI 0.9935-1.1389). Consequently, the prognostic value of albumin was enhanced when it was combined with lactate levels. This prognostic value was particularly evident when albumin and lactate levels were analyzed together as dichotomous variables, revealing a statistically significant difference. In our multivariate regression analysis, the modeling outcomes revealed a negative correlation between LAR and SNL. Following the adjustment for potential confounders, this relationship remained stable. In a stratified analysis, when LAR≥10.5%, the trend corresponded to that observed with the continuous variable, with highly pronounced statistical significance. These results imply that LAR may act as an independent prognostic factor for adverse outcomes in PALF patients.

LAR, initially described by Wang et al. in 2015, has been suggested as a prognostic indicator of mortality and onset of organ dysfunction [33]. Subsequent research has shown a correlation between increased LAR and poor outcomes in various diseases including sepsis, AKI, and pancreatitis [34-36]. A study utilizing the eICU database revealed that LAR was significantly associated with both hospital and ICU mortality (adjusted hazard ratio [HR] 1.22, 95% CI 1.18-1.26, P<0.0001 for both). These findings indicate that elevated LAR is an independent risk factor for in-hospital and intensive care unit (ICU) mortality among critically ill patients with AKI [36].

An additional study that enrolled 648 critically ill children in pediatric intensive care units (PICUs) throughout the United States demonstrated that early implementation of LAR was significantly correlated with mortality and the onset of MODS, compared to the individual initial measurements of lactate or albumin alone [26]. However, studies investigating the relationship between LAR and liver disease are more common in adults, while they are scarce in children. Several studies have shown that LAR is an effective prognostic marker in cirrhotic and ACLF patients. Elevated LAR is significantly associated with in-hospital mortality and liver-related adverse outcomes, with similar prognostic value to traditional scoring systems such as APACHE II and SOFA scores [37-39].

Although most prior studies on LAR have focused on chronic liver disease or critical illness, its use in PALF is pathophysiologically justified. In PALF, LAR likely reflects both acute hepatic dysfunction and systemic stress: elevated lactate suggests impaired clearance and mitochondrial failure, while low albumin indicates reduced synthesis, inflammation, and may also reflect capillary leakage. Unlike in sepsis or ACLF, where LAR reflects circulatory failure, in PALF it may represent overall metabolic crisis and liver reserve. Although a high LAR was inversely associated with SNL, we interpret it broadly as a marker of poor prognosis, rather than distinguishing between death and transplant—consistent with our study’s focus on native liver survival.

We also acknowledge that treatment-related factors prior to or during early PICU admission may influence initial LAR values. For instance, the use of vasoactive agents such as epinephrine can increase lactate levels independently of tissue hypoxia, thereby potentially confounding the association between LAR and clinical outcomes. Although detailed data on drug types and dosages were unavailable, we performed a stratified analysis and found that the association between high LAR and reduced SNL was significant only in the non-shock subgroup. This suggests that LAR is more robust as a prognostic marker when not influenced by shock-related hyperlactatemia.

Furthermore, in the present study, both the GAM and RCS models revealed a linear correlation between LAR and SNL. This implies that in clinical settings, an elevated LAR is associated with a greater likelihood of adverse outcomes, such as heightened mortality or the need for liver transplantation, corroborating the findings of prior studies [35, 36].

In the subgroup analysis, LAR was found to be correlated with SNL in patients who did not experience shock. As is well known, the pathophysiology of shock is characterized by inadequate tissue perfusion, which is a primary contributor to the excessive production of lactate. A significant correlation between the LAR and SNL was evident when the confounding effects of tissue hypoperfusion on lactate levels were excluded. Consequently, shock, with its signature tissue hypoperfusion, has emerged as a significant confounder in the relationship between LAR and SNL. Although we recognize that the constrained sample size within the subgroup analysis may impact the robustness of the findings, these results are congruent with the established pathophysiological mechanisms of the disease.

This study offers robust evidence supporting the relationship between LAR and SNL in patients with PALF, meticulously accounting for potential confounders and bias. However, this study had several limitations. First, this was a single-center retrospective analysis. Some cases were excluded due to missing early albumin data, which may introduce selection bias. To improve data completeness and study robustness, we are actively planning to integrate institutional databases and conduct prospective cohort studies. Second, the study’s limited sample size restricted its ability to conduct a comprehensive evaluation of statistical power and investigate interactions among various factors. Given the rarity of PALF, the small population size represents a significant limitation not only for the current study but also for similar research. Nonetheless, our study successfully enrolled a larger cohort of participants from a single institution than previous studies, and it provides detailed insights into the clinical characteristics of PALF in the Northeast region of China, which have not been previously described. Finally, in order to minimize the influence of confounding variables, we chose to use the initial measurements within the first 24 hours of hospital admission, thereby avoiding the potential impact of subsequent therapeutic interventions on the study outcomes. However, this approach also precludes the assessment of the dynamic effects of LAR changes on clinical prognosis. In future studies, we plan to consider performing multiple lactate measurements at different time points (such as at admission, at 6 hours, peak lactate, and lactate clearance) and use these dynamic changes to calculate the lactate-albumin ratio. This will help provide a more comprehensive evaluation of the clinical significance of the lactate-albumin ratio as a prognostic marker.

## Conclusion

In summary, this study revealed a significant negative correlation between early LAR and SNL in patients with PALF, indicating that early LAR may be a more reliable indicator of SNL than either initial lactate or initial albumin considered in isolation. LAR demonstrated a markedly stronger association with SNL than when initial lactate or albumin levels were evaluated alone. The results of this study have implications for clinical decision-making and the prognostic evaluation of PALF. Acknowledging the potential for confounding factors, additional research is warranted to confirm these findings and deepen our understanding of this relationship.

**Author contributions statement (CRediT taxonomy):**

Conceptualization: Zhen Zhang, Yuna Li, Jingwen Li, Yumei Li.

Methodology: Zhen Zhang, Yuna Li, Jingwen Li, Yumei Li.

Material preparation, data collection, and analysis: Zhen Zhang, Yuna Li, Jingwen Li.

Writing–Original Draft Preparation: Zhen Zhang.

Writing–Review & Editing: Zhen Zhang, Yuna Li, Jingwen Li, Yumei Li.

Approval of Final Manuscript: Zhen Zhang, Yuna Li, Jingwen Li, Yumei Li.

**Funding details:**

This work was supported by the Science Foundation of Jilin Province (Grant No. YDZJ202301ZYTS056) and the Jilin Province Health Science and Technology Capacity Improvement Project (Grant No. 2023JC002). The funders played no role in the study design, data collection, analysis, interpretation, or decision to publish.

**Acknowledgements:**

We extend our heartfelt gratitude to all the participants for their invaluable contributions to this study. Special thanks are due to Dr. Jie Liu from the Department of Vascular and Endovascular Surgery at the Chinese PLA General Hospital for his invaluable assistance in the study design, language editing, proofreading, statistical support, and insightful comments on the manuscript.

**Disclosure statements:**

The authors report no conflict of interest.

**Data Availability Statement:**

The raw data necessary to replicate the findings of this study cannot be shared at this time as they are part of an ongoing study. Upon request, some or all data generated or utilized during the study can be obtained from the corresponding author.

**Ethics approval and consent to participate:**

This study was performed in line with the principles of the Declaration of Helsinki. Approval was granted by the Ethics Committee of the First Hospital of Jilin University (Date 23/07/2024 No 2024-793), which also waived the requirement for informed consent due to the retrospective nature of the study.

## List of abbreviations

ACLF: Acute-on-Chronic Liver Failure;

AKI: acute kidney injury;

ALT: alanine aminotransferase;

AST: aspartate aminotransferase;

BNP: B-type natriuretic peptide;

CRRT: continuous renal replacement therapy;

CIs: confidence intervals;

DBIL: direct bilirubin;

DPMAS: double plasma molecular adsorption system;

DCD: donation after circulatory death

GAM: generalized additive model;

GGT: gamma-glutamyltransferase;

HE: hepatic encephalopathy;

ICU: Intensive Care Units;

INR: international normalized ratio;

IQR: interquartile range;

LAR: lactate-albumin ratio;

LIU: liver injury unit;

LT: liver transplantation;

LDLT: living donor liver transplantation;

MELD: model for end-stage liver disease;

MODS: multiple organ dysfunction syndrome;

NH3: ammonia;

ORs, odds ratios;

PALF, Pediatric acute liver failure;

PALFSG, PALF Study Group;

PICU: pediatric intensive care unit;

PT: prothrombin time;

PE: plasma exchange

RCS: restricted cubic splines;

SNL: survival with the native liver;

TBIL: total bilirubin;

TnI: troponin I;

WBC: white blood cell

## References:

1. Jain V, Dhawan A: **Prognostic modeling in pediatric acute liver failure**. *Liver Transpl* 2016, **22**(10):1418-1430.

2. Squires JE, Alonso EM, Ibrahim SH, Kasper V, Kehar M, Martinez M, Squires RH: **North American Society for Pediatric Gastroenterology, Hepatology, and Nutrition Position Paper on the Diagnosis and Management of Pediatric Acute Liver Failure**. *J Pediatr Gastroenterol Nutr* 2022, **74**(1):138-158.

3. Vento S, Cainelli F: **Acute liver failure in low-income and middle-income countries**. *Lancet Gastroenterol Hepatol* 2023, **8**(11):1035-1045.

4. Dhawan A: **Etiology and prognosis of acute liver failure in children**. *Liver Transpl* 2008, **14 Suppl 2**:S80-84.

5. Karpen SJ: **Acute Hepatitis in Children in 2022 - Human Adenovirus 41?** *N Engl J Med* 2022, **387**(7):656-657.

6. Sabapathy DG, Desai MS: **Acute Liver Failure in Children**. *Pediatr Clin North Am* 2022, **69**(3):465-495.

7. Lenz D, Horby Jorgensen M, Kelly D, Cardinale V, Geerts A, Goncalves Costa I, Fichtner A, Garbade SF, Hegen B, Hilberath J *et al*: **Etiology and Outcome of Adult and Pediatric Acute Liver Failure in Europe**. *J Pediatr Gastroenterol Nutr* 2023, **77**(1):115-120.

8. Lenz D, Schlieben LD, Shimura M, Bianzano A, Smirnov D, Kopajtich R, Berutti R, Adam R, Aldrian D, Baric I *et al*: **Genetic landscape of pediatric acute liver failure of indeterminate origin**. *Hepatology* 2024, **79**(5):1075-1087.

9. Bernal W, Wendon J: **Acute liver failure**. *N Engl J Med* 2013, **369**(26):2525-2534.

10. Reddy KR, Ellerbe C, Schilsky M, Stravitz RT, Fontana RJ, Durkalski V, Lee WM, Acute Liver Failure Study G: **Determinants of outcome among patients with acute liver failure listed for liver transplantation in the United States**. *Liver Transpl* 2016, **22**(4):505-515.

11. Ge J, Kim WR, Lai JC, Kwong AJ: **"Beyond MELD" - Emerging strategies and technologies for improving mortality prediction, organ allocation and outcomes in liver transplantation**. *J Hepatol* 2022, **76**(6):1318-1329.

12. Sundaram V, Shneider BL, Dhawan A, Ng VL, Im K, Belle S, Squires RH: **King's College Hospital Criteria for non-acetaminophen induced acute liver failure in an international cohort of children**. *J Pediatr* 2013, **162**(2):319-323 e311.

13. Edmark C, McPhail MJW, Bell M, Whitehouse T, Wendon J, Christopher KB: **LiFe: a liver injury score to predict outcome in critically ill patients**. *Intensive Care Med* 2016, **42**(3):361-369.

14. Bernal W, Hyyrylainen A, Gera A, Audimoolam VK, McPhail MJ, Auzinger G, Rela M, Heaton N, O'Grady JG, Wendon J *et al*: **Lessons from look-back in acute liver failure? A single centre experience of 3300 patients**. *J Hepatol* 2013, **59**(1):74-80.

15. European Association for the Study of the Liver. Electronic address eee, Clinical practice guidelines p, Wendon J, Panel m, Cordoba J, Dhawan A, Larsen FS, Manns M, Samuel D, Simpson KJ *et al*: **EASL Clinical Practical Guidelines on the management of acute (fulminant) liver failure**. *J Hepatol* 2017, **66**(5):1047-1081.

16. Ascher-Bartlett JM, Bangerth S, Jordan S, Weaver C, Barhouma S, Etesami K, Kohli R, Emamaullee J: **CHALF Score: A Novel Tool to Rapidly Risk Stratify Children in Need of Liver Transplant Evaluation During Acute Liver Failure**. *Transplantation* 2024, **108**(4):930-939.

17. Walabh P, Meyer A, de Maayer T, Moshesh PN, Hassan IE, Walabh P, Hajinicolaou C: **Prognostic factors and scoring systems associated with outcome in pediatric acute liver failure**. *BMC Pediatr* 2022, **22**(1):516.

18. Pendergrast TR, Chapin CA, Kriegermeier AA, Pardo AC, Bass LM, Sanchez-Pinto LN: **Heart rate variability is associated with encephalopathy and outcomes in pediatric acute liver failure**. *Pediatr Res* 2023, **93**(5):1348-1353.

19. Gao Y, Zhang H, Zhong H, Yang S, Wang Q: **Lactate and blood ammonia on admission as biomarkers to predict the prognosis of patients with acute mushroom poisoning and liver failure: a retrospective study**. *Toxicol Res (Camb)* 2021, **10**(4):850-855.

20. Woreta TA, Alqahtani SA: **Evaluation of abnormal liver tests**. *Med Clin North Am* 2014, **98**(1):1-16.

21. Sharma N, Pandey S, Yadav M, Mathew B, Bindal V, Sharma N, Tripathi G, Bhat SH, Gupta A, Maiwall R *et al*: **Biomolecular map of albumin identifies signatures of severity and early mortality in acute liver failure**. *J Hepatol* 2023, **79**(3):677-691.

22. Ma Y, Du L, Bai L, Tang H: **Association between lactate-to-albumin ratio and short-term prognosis of acute-on-chronic liver failure treated with artificial liver support system**. *Eur J Gastroenterol Hepatol* 2025, **37**(3):327-336.

23. Manosalva C, Quiroga J, Hidalgo AI, Alarcon P, Anseoleaga N, Hidalgo MA, Burgos RA: **Role of Lactate in Inflammatory Processes: Friend or Foe**. *Front Immunol* 2021, **12**:808799.

24. O'Brien C, Beaubien-Souligny W, Amsallem M, Denault A, Haddad F: **Cardiogenic Shock: Reflections at the Crossroad Between Perfusion, Tissue Hypoxia, and Mitochondrial Function**. *Can J Cardiol* 2020, **36**(2):184-196.

25. Caironi P, Langer T, Gattinoni L: **Albumin in critically ill patients: the ideal colloid?** *Curr Opin Crit Care* 2015, **21**(4):302-308.

26. Ray CC, Pollack MM, Gai J, Patel AK: **The Association of the Lactate-Albumin Ratio With Mortality and Multiple Organ Dysfunction in PICU Patients**. *Pediatr Crit Care Med* 2023, **24**(9):760-766.

27. Shadvar K, Nader-Djalal N, Vahed N, Sanaie S, Iranpour A, Mahmoodpoor A, Vahedian-Azimi A, Samim A, Rahimi-Bashar F: **Comparison of lactate/albumin ratio to lactate and lactate clearance for predicting outcomes in patients with septic shock admitted to intensive care unit: an observational study**. *Sci Rep* 2022, **12**(1):13047.

28. Vandenbroucke JP, von Elm E, Altman DG, Gotzsche PC, Mulrow CD, Pocock SJ, Poole C, Schlesselman JJ, Egger M, Initiative S: **Strengthening the Reporting of Observational Studies in Epidemiology (STROBE): explanation and elaboration**. *Int J Surg* 2014, **12**(12):1500-1524.

29. Ng RT, Chew KS, Choong CL, Song ZL, Teh JKL, Koay ZL, Wong SY, Kam CC, Ranai NBM, Lee WS: **Etiology, outcome and prognostic indicators of acute liver failure in Asian children**. *Hepatol Int* 2022, **16**(6):1390-1397.

30. Kidney Disease: Improving Global Outcomes CKDWG: **KDIGO 2024 Clinical Practice Guideline for the Evaluation and Management of Chronic Kidney Disease**. *Kidney Int* 2024, **105**(4S):S117-S314.

31. Dellinger RP, Levy MM, Rhodes A, Annane D, Gerlach H, Opal SM, Sevransky JE, Sprung CL, Douglas IS, Jaeschke R *et al*: **Surviving Sepsis Campaign: international guidelines for management of severe sepsis and septic shock, 2012**. *Intensive Care Med* 2013, **39**(2):165-228.

32. Bhakta D, Patel M, Ma TW, Boutte J, Sarmast N, Asrani SK: **Model for End-Stage Liver Disease Lactate Score and Prediction of Inpatient Mortality in Critically Ill Patients With Cirrhosis**. *Liver Transpl* 2021, **27**(12):1861-1864.

33. Wang B, Chen G, Cao Y, Xue J, Li J, Wu Y: **Correlation of lactate/albumin ratio level to organ failure and mortality in severe sepsis and septic shock**. *J Crit Care* 2015, **30**(2):271-275.

34. Liu Q, Zheng HL, Wu MM, Wang QZ, Yan SJ, Wang M, Yu JJ, Li DP: **Association between lactate-to-albumin ratio and 28-days all-cause mortality in patients with acute pancreatitis: A retrospective analysis of the MIMIC-IV database**. *Front Immunol* 2022, **13**:1076121.

35. Chen S, Guan S, Yan Z, Ouyang F, Li S, Liu L, Zhong J: **The lactate to albumin ratio linked to all-cause mortality in critically ill patients with septic myocardial injury**. *Front Cardiovasc Med* 2023, **10**:1233147.

36. Zhu X, Xue J, Liu Z, Dai W, Xu H, Zhou Q, Zhao S, Zhou Q, Chen W: **The Lactate/Albumin Ratio Predicts Mortality in Critically Ill Patients with Acute Kidney Injury: An Observational Multicenter Study on the eICU Database**. *Int J Gen Med* 2021, **14**:10511-10525.

37. Krispin I, Mahamid M, Goldin E, Fteiha B: **Elevated lactate/albumin ratio as a novel predictor of in-hospital mortality in hospitalized cirrhotics**. *Ann Hepatol* 2023, **28**(3):100897.

38. Golcuk Y, Golcuk BK: **Prognostic value of lactate/albumin ratio in patients with acute-on-chronic liver failure**. *Ann Hepatol* 2023, **28**(6):101161.

39. Boyaci Dundar N, Inci K, Turkoglu M, Aygencel G: **Comparison of lactate/albumin ratio and established scoring systems for predicting mortality in critically ill cirrhotic patients**. *Rev Esp Enferm Dig* 2024, **116**(10):539-545.
